# 4-Ammonio­benzamidinium dichloride

**DOI:** 10.1107/S1600536808010179

**Published:** 2008-05-03

**Authors:** Y. M. Legrand, A. van der Lee, M. Barboiu

**Affiliations:** a Institut Européen des Membranes, UMR 5635, CC 047 Université de Montpellier II, Montpellier, France

## Abstract

The crystal structure of the title compound, C_7_H_11_N_3_^2+^·2Cl^−^, has been determined as part of a project focusing on the ability of the benzamidine system to form strong hydrogen bonds in aqueous media. It is commonly used as a ligand in affinity chromatography for purification and immobilization of enzymes. A twofold rotation axis runs along the axis of the cation. The orientation of the amidinium group with respect to the benzene ring is indicated by the N—C—C—C torsion angle of 40.2 (1)°. In the crystal structure, cations and anions are linked *via* hydrogen bonds. The chloride anion is surrounded by four ammonium cations in a tetra­hedral environment. The aromatic rings of the amidinium cations are π-stacked, with a centroid–centroid distance of 4.178 (1) Å.

## Related literature

For related literature, see: Boyd (1991 ▶); Nguyen & Loung (1990 ▶); Jarak *et al.* (2005 ▶); Hranjec *et al.* (2003 ▶); Danan *et al.* (1997 ▶); Del Poeta, Schell, Dykstra, Jones, Tidwell, Czarny *et al.* (1998 ▶); Del Poeta, Schell, Dykstra, Jones, Tidwell, Kumar *et al.*, (1998 ▶); Janiak (2000 ▶); Fujita *et al.* (1995 ▶); Müller *et al.* (2006 ▶); Kimata *et al.* (1990 ▶). For examples of related tubular superstructures, see: Barboiu *et al.* (2003 ▶); Blondeau *et al.* (2005 ▶).
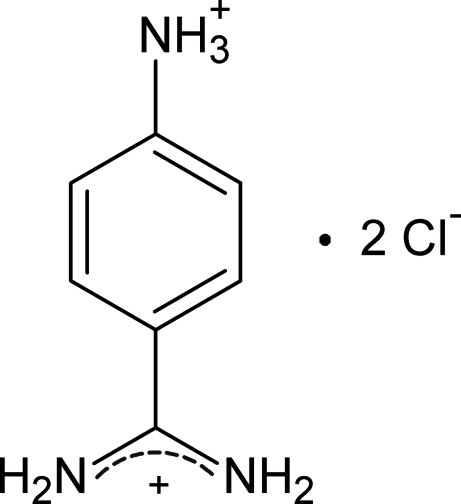


## Experimental

### 

#### Crystal data

C_7_H_11_N_3_^2+^·2Cl^−^
*M* *_r_* = 208.09
Monoclinic, 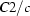

*a* = 4.1779 (2) Å
*b* = 20.9388 (10) Å
*c* = 11.6260 (5) Å
β = 94.920 (4)°
*V* = 1013.30 (8) Å^3^
*Z* = 4
Mo *K*α radiation
μ = 0.59 mm^−1^
*T* = 175 K
0.49 × 0.09 × 0.05 mm

#### Data collection

Oxford Diffraction XCalibur diffractometer
Absorption correction: multi-scan (*CrysAlis RED*; Oxford Diffraction, 2007 ▶) *T*_min_ = 0.95, *T*_max_ = 0.97
7752 measured reflections
1750 independent reflections
1144 reflections with *I* > 2σ(*I*)
*R*_int_ = 0.018

#### Refinement

*R*[*F* ^2^ > 2σ(*F* ^2^)] = 0.027
*wR*(*F* ^2^) = 0.034
*S* = 1.00
1144 reflections
59 parameters
2 restraints
H-atom parameters constrained
Δρ_max_ = 0.34 e Å^−3^
Δρ_min_ = −0.20 e Å^−3^

### 

Data collection: *CrysAlis CCD* (Oxford Diffraction, 2007 ▶); cell refinement: *CrysAlis RED* (Oxford Diffraction, 2007 ▶); data reduction: *CrysAlis RED*; program(s) used to solve structure: *SIR2004* (Burla *et al.*, 2003 ▶); program(s) used to refine structure: *CRYSTALS* (Betteridge *et al.*, 2003 ▶); molecular graphics: *CAMERON* (Watkin *et al.*, 1996 ▶) and *Mercury* (Macrae *et al.*, 2006 ▶); software used to prepare material for publication: *CRYSTALS*.

## Supplementary Material

Crystal structure: contains datablocks global, I. DOI: 10.1107/S1600536808010179/wn2252sup1.cif

Structure factors: contains datablocks I. DOI: 10.1107/S1600536808010179/wn2252Isup2.hkl

Additional supplementary materials:  crystallographic information; 3D view; checkCIF report

## Figures and Tables

**Table 1:** Hydrogen-bond geometry (Å, °)

*D*—H⋯*A*	*D*—H	H⋯*A*	*D*⋯*A*	*D*—H⋯*A*
N2—H9⋯Cl1^i^	0.90	2.35	3.2247 (13)	166
N2—H10⋯Cl1	0.92	2.32	3.2142 (13)	162
N8—H13⋯Cl1^ii^	0.85	2.26	3.1031 (6)	173
N8—H14⋯Cl1^iii^	0.94	2.20	3.1369 (6)	176
C5—H11⋯Cl1^iv^	1.00	2.70	3.6806 (13)	165

Symmetry codes: (i) 
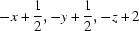
; (ii) 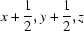; (iii) 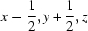; (iv) 
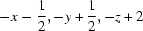
.

**Fig. 1.**
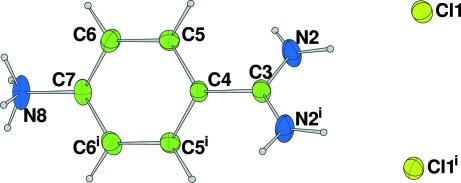
Representation of the structure of the title compound, with the numbering scheme adopted. The Cl atoms is light-green, the C atom green, the N atoms blue and the H atoms in grey. Displacement ellipsoids are drawn at the 50% probability level [symmetry code: (i) -x, y, -z + 3/2].

**Fig. 2.**
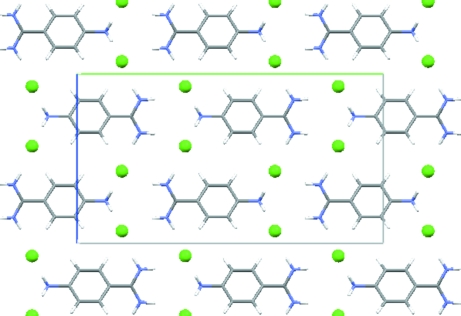
The two-dimensional framework of the title compound, viewed down the a cell direction.

**Fig. 3.**
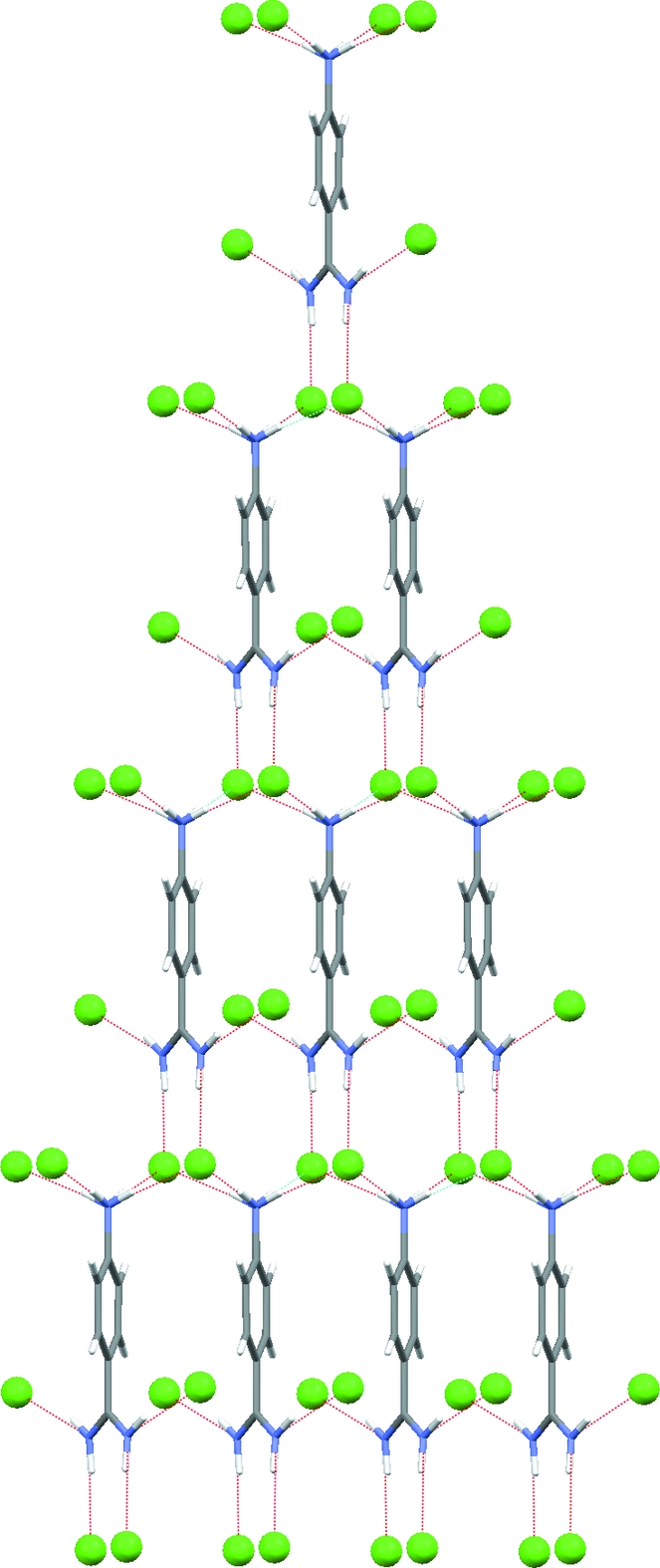
Representation of the hydrogen bonding network between the cations and the chloride anions, giving rise to a pyramidal scaffold architecture. Hydrogen bonds are denoted by dotted lines.

## supplementary crystallographic information

### Comment

Several types of heteroditopic receptors, including the title compound, are being used in our group as bricks for supramolecular construction (Barboiu *et al.*, 2003, Blondeau *et al.*, 2005). Among other functions, amidine compounds have shown antiparasitic (Danan *et al.*, 1997) and antifungal activity (Del Poeta, Schell, Dykstra, Jones, Tidwell, Czarny *et al.*, 1998; Del Poeta, Schell, Dykstra, Jones, Tidwell, Kumar *et al.*, 1998). Indeed, this class of compounds has been widely studied for its biological activities. Surprisingly, only one crystal structure of an aminobenzamidine derivative has been published so far (Jarak *et al.*, 2005). Our project deals with the construction of supramolecular architectures based on hydrogen bonding in aqueous media. This is possible due to the strength of the bonds formed between the very electrophilic amidinium unit and the nucleophilic character of acids, for example. Superstructures made of co-crystals have been designed and surprising results have been achieved by Fujita *et al.* (1995) and Müller *et al.* (2006). The amidine group also forms a well recognized class of anticancer compounds (Boyd, 1991, Hranjec *et al.*, 2003) and, based on the same properties, can also be used as ligands in affinity chromatography to immobilize enzymes (Nguyen & Loung, 1990 and Kimata *et al.*, 1990).

The molecule of the title compound (Fig. 1) is not planar. The amidinium group has a synclinal disposition with respect to the benzene ring (N2—C3—C4—C5 = -40.2 (1)°). A twofold rotation axis runs along the axis of the cation. The observed deviation from coplanarity might serve to accommodate the formation of intermolecular hydrogen bonds with chloride ions. The three ammonium protons are free to rotate about the C7—N8 bond. These protons were found by Fourier difference maps at four positions (2 + 2 by symmetry) which appears to be in line with the four chloride anions surrounding the ammonium group (N···Cl = 3.103 Å). The site occupation factors of the four ammonium protons was set at 0.75, as there are, in fact, only three protons attached to this ammonium nitrogen. As Fig. 2 shows, rows of head-to-tail benzamidine are stacked alternately. Interestingly, three of four nitrogen atoms form a plane on which the chloride atom sits, almost perfectly. Each chloride anion is bound to four nitrogen cations by weak hydrogen bonds (N···Cl = 3.103 (1)–3.225 (1) Å), while each amidinium unit is bound to eight chloride anions (four times through the ammonium site, twice through each amidinium nitrogen). This produces a singular pyramidal architecture, as depicted in Fig. 3. The packing is determined by these hydrogen bonds, but also by π-stacking. The aromatic rings of the amidinium cations adopt a parallel offset conformation. The distance *Cg*···*Cg* between the centroids of two adjacent rings is 4.178 (1) Å, whereas the angle between the ring-centroid vector and the ring normal of one of the amidine rings is 27.7 (1)° (with a perpendicular interplanar distance of 3.7 Å). The angle between the two benzene rings is 0.02°. These values can be considered to be normal for π-π interactions (Janiak, 2000). Fig. 3 also shows both the hydrogen bonding pattern and the interactions between the aromatic groups held together by π-π non-covalent intermolecular interactions.

### Experimental

The title compound is commercially available. To purify it, it has been crystallized from a mixture of water and methanol (10:2). The crystals formed over a period of one week.

### Refinement

The H atoms, including those attached to nitrogen atoms, were all located in a difference map, and then repositioned geometrically. The H atoms were initially refined with soft restraints on the bond lengths and angles to regularize their geometry (C—H in the range 0.99-1.00, N—H = 0.85-0.94 Å) and *U*_iso_(H) (in the range 1.3-1.8 times *U*_eq_ of the parent atom), after which the positions were refined with riding constraints.

### Crystal data

**Table:**

C_7_H_11_N_3_^2+^·2Cl^–^	*F*_000_ = 432
*M**_r_* = 208.09	*D*_x_ = 1.364 Mg m^−^^3^
Monoclinic, *C*2/*c*	Mo *K*α radiation λ = 0.71073 Å
Hall symbol: -C 2yc	Cell parameters from 4424 reflections
*a* = 4.1779 (2) Å	θ = 4–32º
*b* = 20.9388 (10) Å	µ = 0.59 mm^−^^1^
*c* = 11.6260 (5) Å	*T* = 175 K
β = 94.920 (4)º	Stick, colourless
*V* = 1013.30 (8) Å^3^	0.49 × 0.09 × 0.05 mm
*Z* = 4	

### Data collection

**Table:**

Oxford Diffraction XCALIBUR diffractometer	1750 independent reflections
Radiation source: Enhance (Mo) X-ray Source	1144 reflections with *I* > 2σ(*I*)
Monochromator: graphite	*R*_int_ = 0.018
Detector resolution: 16.0143 pixels mm^-1^	θ_max_ = 32.7º
*T* = 175 K	θ_min_ = 3.9º
ω scans	*h* = −5→6
Absorption correction: multi-scan(CrysAlis RED; Oxford Diffraction, 2007); empirical (using intensity measurements) absorption correction using spherical harmonics, implemented in SCALE3 ABSPACK scaling algorithm	*k* = −31→29
*T*_min_ = 0.95, *T*_max_ = 0.97	*l* = −17→15
7752 measured reflections	

### Refinement

**Table:**

Refinement on *F*	Primary atom site location: structure-invariant direct methods
Least-squares matrix: full	Hydrogen site location: inferred from neighbouring sites
*R*[*F*^2^ > 2σ(*F*^2^)] = 0.027	H-atom parameters constrained
*wR*(*F*^2^) = 0.034	Method, part 1, Chebychev polynomial [Watkin, D. J. (1994). Acta Cryst. A50, 411-437. *P*rince, E. (1982). Mathematical Techniques in Crystallography and Materials Science. Ne*w* York: Springer-Verlag.] [*w*eight] = 1.0/[A_0_*T_0_(x) + A_1_*T_1_(x) ··· + A_n-1_]*T_n-1_(x)] where A_i_ are the Chebychev coefficients listed belo*w* and x = *F* /*F*max Method = Robust Weighting (*P*rince, 1982) W = [*w*eight] * [1-(delta*F*/6*sigma*F*)^2^]^2^ A_i_ are 20.0 -14.7 15.4
*S* = 1.01	(Δ/σ)_max_ = 0.001
1144 reflections	Δρ_max_ = 0.34 e Å^−^^3^
59 parameters	Δρ_min_ = −0.20 e Å^−^^3^
2 restraints	Extinction correction: None

### Fractional atomic coordinates and isotropic or equivalent isotropic displacement parameters (Å^2^)

**Table:**

	*x*	*y*	*z*	*U*_iso_*/*U*_eq_	Occ. (<1)
Cl1	0.03602 (8)	0.146532 (14)	0.92627 (3)	0.0297	
N2	0.0839 (4)	0.29292 (6)	0.84632 (10)	0.0380	
C3	0.0000	0.32303 (8)	0.7500	0.0271	
C4	0.0000	0.39368 (8)	0.7500	0.0236	
C5	−0.1109 (3)	0.42664 (6)	0.84251 (11)	0.0282	
C6	−0.1138 (3)	0.49282 (6)	0.84200 (11)	0.0296	
C7	0.0000	0.52485 (8)	0.7500	0.0264	
N8	0.000000 (10)	0.59418 (7)	0.750000 (10)	0.0381	
H9	0.1600	0.3149	0.9089	0.0500*	
H10	0.0659	0.2491	0.8520	0.0500*	
H11	−0.1867	0.4022	0.9091	0.0500*	
H12	−0.1892	0.5181	0.9058	0.0500*	
H13	0.1342	0.6109	0.8006	0.0500*	0.7500
H14	−0.1333	0.6116	0.8033	0.0500*	0.7500

### Atomic displacement parameters (Å^2^)

**Table:**

	*U*^11^	*U*^22^	*U*^33^	*U*^12^	*U*^13^	*U*^23^
Cl1	0.04130 (17)	0.02485 (14)	0.02214 (13)	−0.00435 (13)	−0.00233 (9)	−0.00073 (11)
N2	0.0652 (9)	0.0209 (5)	0.0252 (5)	−0.0030 (5)	−0.0122 (5)	0.0038 (4)
C3	0.0386 (9)	0.0198 (7)	0.0218 (7)	0.0000	−0.0043 (6)	0.0000
C4	0.0324 (9)	0.0184 (6)	0.0191 (6)	0.0000	−0.0030 (6)	0.0000
C5	0.0403 (7)	0.0236 (5)	0.0208 (5)	0.0006 (5)	0.0021 (4)	0.0000 (4)
C6	0.0379 (7)	0.0238 (6)	0.0265 (5)	0.0036 (5)	−0.0011 (4)	−0.0043 (4)
C7	0.0275 (8)	0.0180 (6)	0.0319 (8)	0.0000	−0.0074 (6)	0.0000
N8	0.0296 (8)	0.0177 (7)	0.0651 (12)	0.0000	−0.0072 (8)	0.0000

### Geometric parameters (Å, °)

**Table:**

N2—C3	1.3064 (13)	C6—C7	1.3810 (16)
N2—H9	0.896	C6—H12	0.985
N2—H10	0.923	C7—N8	1.452 (2)
C3—C4	1.479 (2)	N8—H14^i^	0.942
C4—C5	1.3904 (15)	N8—H13^i^	0.852
C5—C6	1.3859 (18)	N8—H13	0.852
C5—H11	1.002	N8—H14	0.942
			
C3—N2—H9	120.0	C6—C7—N8	119.05 (8)
C3—N2—H10	121.5	C6^i^—C7—N8	119.05 (8)
H9—N2—H10	118.5	C7—N8—H14^i^	112.8
N2—C3—N2^i^	122.30 (16)	C7—N8—H13^i^	114.3
N2—C3—C4	118.85 (8)	H14^i^—N8—H13^i^	77.2
C3—C4—C5	119.75 (8)	C7—N8—H13	114.3
C5^i^—C4—C5	120.49 (16)	H14^i^—N8—H13	84.5
C4—C5—C6	119.76 (13)	H13^i^—N8—H13	131.5
C4—C5—H11	119.5	C7—N8—H14	112.8
C6—C5—H11	120.7	H14^i^—N8—H14	134.3
C5—C6—C7	119.04 (13)	H13^i^—N8—H14	84.5
C5—C6—H12	122.5	H13—N8—H14	77.2
C7—C6—H12	118.5		
			
C(4)—C(5)—C(6)—C(7)	−1.2 (1)	C(5)—C(6)—C(7)—N(8)	−179.4 (1)
C(5)—C(4)—C(3)—N(2)	−40.2 (1)		

Symmetry codes: (i) −*x*, *y*, −*z*+3/2.

### Hydrogen-bond geometry (Å, °)

**Table:**

*D*—H···*A*	*D*—H	H···*A*	*D*···*A*	*D*—H···*A*
N2—H9···Cl1^ii^	0.90	2.35	3.2247 (13)	166
N2—H10···Cl1	0.92	2.32	3.2142 (13)	162
N8—H13···Cl1^iii^	0.85	2.26	3.1031 (6)	173
N8—H14···Cl1^iv^	0.94	2.20	3.1369 (6)	176
C5—H11···Cl1^v^	1.00	2.70	3.6806 (13)	165

Symmetry codes: (ii) −*x*+1/2, −*y*+1/2, −*z*+2; (iii) *x*+1/2, *y*+1/2, *z*; (iv) *x*−1/2, *y*+1/2, *z*; (v) −*x*−1/2, −*y*+1/2, −*z*+2.
